# The Impact of Respiratory Symptoms on the Risk of Serious Bacterial Infection in Febrile Infants < 60 Days Old

**DOI:** 10.3390/jcm12144636

**Published:** 2023-07-12

**Authors:** Kamal Masarweh, Lea Bentur, Ronen Bar-Yoseph, Imad Kassis, Halima Dabaja-Younis, Michal Gur

**Affiliations:** 1 Pediatric Pulmonary Institute, CF Center, Rappaport Children’s Hospital, Rambam Health Care Campus, Haifa 3109601, Israel; m_kamal@rambam.health.gov.il (K.M.); l_bentur@rambam.health.gov.il (L.B.); r_bar-yoseph@rambam.health.gov.il (R.B.-Y.); 2 Rappaport Faculty of Medicine, Technion—Israel Institute of Technology, Haifa 3109601, Israel; i_kassis@rambam.health.gov.il (I.K.); haleemayounis1982@gmail.com (H.D.-Y.); 3 Pediatric Infectious Diseases Unit, Rappaport Children’s Hospital, Haifa 3109601, Israel; 4 Department of Pediatrics B, Rappaport Children’s Hospital, Rambam Health Care Campus, Haifa 3109601, Israel

**Keywords:** respiratory symptoms, serious bacterial infection, febrile infants

## Abstract

Objectives: We aimed to evaluate the impact of respiratory symptoms and positive viral testing on the risk of serious bacterial infections (SBIs). Methods: A retrospective study was conducted that included infants (0–60 days) presenting with a fever between 2001 and 2022 at a tertiary hospital in northern Israel. Demographic, clinical, and laboratory parameters were collected, and risk factors for SBIs were analyzed. Results: Data from a total of 3106 infants, including data from blood, urine, and CSF cultures, were obtained in 96.6%, 89%, and 29% of cases, respectively. A fever without respiratory symptoms (fever only) was present in 1312 infants, while 1794 had a fever and respiratory symptoms—427 were positive for a respiratory virus (virus+), 759 tested negative (virus−), and 608 were not tested. The SBI rate was 5.1% vs. 7.5% in the fever-and-respiratory group vs. the fever-only group (*p* = 0.004, OR = 0.65 (95% CI = 0.49–0.88)) and 2.8% vs. 7% in the virus+ vs. virus− group (*p* = 0.002, OR = 0.385, (95% CI = 0.203–0.728)). The male gender, an age < 1 month, leukocytosis > 15 × 10^9^/L, or a CRP > 2 mg/dL increased the risk of SBIs. Respiratory symptoms or a confirmed viral infection reduced the risk of SBIs in the presence of the above risk factors. Conclusions: Respiratory symptoms and a positive viral test decreased the risk of SBIs. Combining rapid viral testing with clinical variables may identify low-risk infants. Despite the relatively low risk of SBIs in individuals with viral infections, conducting prospective studies remains essential for accurately predicting the occurrence of these potentially life-threatening infections.

## 1. Introduction

The incidence of serious bacterial infections (SBIs), defined as a urinary tract infection (UTI), bacteremia, or acute bacterial meningitis, ranges from 8% to 13% in young infants presenting at a hospital with a fever [1]. The relatively immature immune system and lack of vaccination of these young infants predispose them to developing invasive bacterial illnesses [2]. This has prompted physicians to perform extensive evaluations, hospitalizations, and antimicrobial treatments on most febrile infants younger than 60 days [3]. Due to the concerns of complications resulting from hospitalizing such young infants, a tremendous effort has been made in the last decades to develop clinical strategies for more selective management and hospitalizations [4]. In 2021, the American Academy of Pediatrics (AAP) released clinical practice guidelines for the management of young febrile infants. The guidelines recommend a full sepsis workup (blood, urine, and cerebrospinal fluid (CSF) cultures), the administration of empirical parenteral antibiotics, and hospitalization for all febrile infants aged 8 to 21 days old; more selective management is suggested for older infants according to clinical and laboratory tests [5].

A challenging group of infants are those with respiratory symptoms, as evidenced by the heterogeneous approach of various studies. For example, the latest AAP guidelines suggest the inclusion of infants with respiratory symptoms in the suggested pathways, but not those with typical symptoms of acute bronchiolitis [5].

A similar conundrum is related to the use of respiratory viral testing. Although previous studies have found a lower risk of SBIs in patients with a proven viral infection, such as respiratory syncytial virus (RSV) or influenza, the risk is not negligible; hence, convincing data to change decision-making strategies are not yet available [6,7]. The current AAP guidelines suggest considering an individualized approach in the management of infants older than 28 days of age with a positive viral test and highlight the need for more research to guide the incorporation of multiplex viral testing into prediction models and guidelines.

Recent advances in the field of data acquisition from medical records allow for the easy and quick retrieval of a large amount of data. Our hospital adopted a new platform named MDClone, a query tool that provides comprehensive patient-level data with a variety of variables in a defined period around an index event. It provides a unique method of retrieving retrospective data on a large number of patients in a quick and computerized manner.

In this study, we aimed to evaluate the effect of respiratory symptoms or a laboratory-confirmed viral infection on the risk of SBIs in febrile infants aged 60 days and younger. Additionally, we aimed to examine the impact on severity markers (including the hospital length of stay (LOS) and pediatric intensive care unit (PICU) admission). Such information can help clinicians with the risk stratification for SBIs in febrile young infants with or without respiratory symptoms and/or confirmed viral pathogens, in addition to the clinical judgement.

## 2. Methods

### 2.1. Study Design

This was a retrospective single tertiary-center study that included all infants 60 days of age and younger who visited our hospital with a diagnosis of a fever between 1 January 2001 and 28 February 2022. The hospital is a 1000-bed (of which 130 are pediatric) tertiary university hospital, with a catchment area of approximately two million heterogeneous citizens (urban/rural; Jews/Arabs; variable socioeconomic status) in northern Israel.

We included infants aged 60 days and younger with a documented temperature ≥ 38 °C at home or upon admission to the emergency department (ED). Data were collected from the patients’ files using the MDClone query tool. The study was approved by the institutional review board (RMB-21-0177).

The data included the following variables:

- Demographics: gender, age on admission, ethnicity, and Arab or Jew.
- Clinical data: presence of respiratory symptoms upon presentation (e.g., cough, wheezing, rhinorrhea) and clinical appearance (ill/appearing well) upon admission (judged by the ED physician upon presentation).

Laboratory studies:

- Microbiological studies:
  - Viral polymerase chain reaction (PCR) for the following viruses: RSV, influenza A, influenza B, parainfluenza, human metapneumovirus (HMPV), adenovirus, rhinovirus, and SARS-CoV-2.
  - Blood cultures.
  - Urine cultures.
  - CSF cultures.
- Blood tests (taken on admission):
- White blood cell count (WBC); leukocytosis was defined as a WBC > 15,000/µL. C-reactive protein (CRP); a high CRP was defined as >2 mg/dL.
- Outcome: The main outcome was the diagnosis of SBIs based on microbiological reports. SBIs were defined by a diagnosis of bacteremia (positive blood culture), meningitis (positive CSF culture), or a UTI (urine culture with >10,000 colony-forming units/mL of uropathogens from a catheterized specimen or any growth from a supra-pubic aspiration [8]). The incidence of an invasive bacterial infection (IBI), defined as bacteremia or meningitis, was also examined. Blood and CSF cultures with the growth of commensal bacteria (e.g., *coagulase-negative staphylococci*, *viridans streptococci*, etc.) when the patient was not treated with antibiotics were considered contaminated.

Specimens for viral testing were collected from the patient’s pharynx and nostrils into sterile viral transport media and transferred immediately to the laboratory or stored at 4 °C for no longer than 2 days. Each sample was tested in parallel in three wells on a PCR plate for influenza A, influenza B, and the internal control (IC) multiplex PCR. The ICs were reviewed annually using external quality-control molecular diagnostics to assess their quality. 

We compared the risk of SBIs between febrile infants without respiratory symptoms (fever only) and febrile infants with respiratory symptoms (fever and respiratory). We then compared the risk of SBIs between infants with a laboratory-confirmed positive viral test (virus+) and a laboratory-confirmed negative viral test (virus−) within the fever-and-respiratory group.

To ensure a homogeneous group of healthy infants without any pre-existing heightened risk of SBIs, infants with underlying conditions or prematurity (gestational age < 37 weeks) were excluded from the study. The exclusion of infants with underlying conditions or prematurity was in line with the recent guidelines from the AAP for the management of febrile infants aged 8–60 days [5].

### 2.2. Statistical Methods

Data were analyzed using SPSS software (version 26). A univariate analysis was performed using the χ^2^ test or Fisher’s exact test. The tests were used to assess the correlation between potential risk factors/predictors (e.g., sex, age, ethnicity, and laboratory measurements) and SBIs in the different groups (fever only versus fever and respiratory symptoms, and virus-negative versus virus-positive) and to examine the differences in the prevalence of SBIs, IBIs, bacteremia, meningitis, and UTIs among the groups (fever only, fever and respiratory symptoms—virus not examined, fever and respiratory symptoms—virus-negative, and fever and respiratory symptoms—virus-positive). The magnitude of the association among these variables was approximated by calculating the crude odds ratios (ORs) and 95% confidence intervals (CIs). All statistical tests were 2-tailed; *p* < 0.05 was considered statistically significant (95% confidence interval). Binary logistic regression was used for the multivariate analysis. Variables with *p* < 0.05 in the univariate analysis were included in the binary regression. The association was considered significant if its coefficient in the binary regression equation remained significant at *p* < 0.05. Adjusted ORs and 95% CIs are presented for the multivariate analysis.

Graphical representations are provided based on boxplots, with the quartiles depicting groups of numerical data and the whiskers indicating variability outside the upper and lower quartiles. A two-sided *p*-value < 0.05 was considered to define statistical significance between the groups. 

## 3. Results

From January 2001 to February 2022, 3106 infants (aged 0–60 days) were discharged from our hospital (ED or pediatric wards) with a diagnosis of a fever. Blood, urine, and CSF cultures were obtained in 96.6%, 89%, and 29% of cases, respectively. Among those without a blood culture available, 85 infants (81%) were older than 28 days, and 82 (78.1%) exhibited respiratory symptoms. The reasons for the absence of a blood culture included a missing reported culture, parental refusal, a lack of a fever upon arrival at the emergency department, and uncertainty regarding the measured fever at home. However, all these infants were monitored until the recovery of symptoms, and none returned to our hospital due to SBIs. 

A fever without respiratory symptoms was present in 1312 (42.2%) infants (fever-only group), while 1794 (57.8%) had a fever accompanied by respiratory symptoms: 427 (23.8%) with a laboratory-confirmed viral infection (virus+), 759 (42.3%) with negative viral testing (virus−), and 608 (33.9%) that were not tested (virus NA). 

Overall, there were 190 cases (6.1%) of SBIs. The rate of SBIs was the lowest in infants with a positive viral test (2.8%) and the highest in infants with a fever only or a fever and respiratory symptoms (virus−), at 7.5% and 7%, respectively (*p* = 0.001). Notably, some infants with SBIs had more than one diagnosis, i.e., meningitis and bacteremia or bacteremia and a UTI. 

IBIs were detected in only two infants with a positive virus test; the first was 12 days old and the other was 21 days old. They both appeared well, but because of their young age, they underwent a full sepsis workup and were hospitalized with antibiotic treatment. The CRP was normal in both. The first patient had normal CSF indices, but a CSF culture grew *Staphylococcus aureus* (*S. aureus*). Eventually, the infant was discharged without further antibiotic treatment. In the second case, the blood count was normal (6400/µL leukocytes), and the blood culture grew *Hemophilus influenza*; despite the good clinical condition and the normal laboratory indices, true bacteremia was assumed, and the child was treated with a targeted antibiotic treatment (Table 1).

In a sub-analysis of infants younger than 28 days (i.e., each week of age analyzed separately), there were no notable differences in the prevalence of SBIs between infants presenting solely with a fever and those exhibiting both a fever and respiratory symptoms (*p* = 0.565 and 0.088, respectively); this finding remained consistent across all four subgroups (*p* = 0.565, *p* = 0.083, *p* = 0.189, and *p* = 0.733 for a fever only and for a fever and respiratory symptoms with virus NA, virus (−), and virus (+), respectively) (Supplementary Materials, Table S1). 

In the univariate and multivariate analyses of risk factors for SBIs, the male gender, an age < 1 months, leukocytosis, an elevated CRP, and a lack of respiratory symptoms increased the risk of SBIs. In the multivariate analysis, all except the male gender remained statistically significant (Table 2).

When comparing febrile infants with and without respiratory symptoms, the rate of SBIs was lower in infants with a fever and respiratory symptoms compared to those with a fever only (5.1% vs. 7.5%, *p* = 0.004, OR = 0.65 (95% CI = 0.49–0.88)). In male infants, infants aged < 1 month, infants that appeared to be well, Jews, and infants with leukocytosis > 15 × 10^9^/L or a CRP > 2 mg/dL, the presence of respiratory symptoms reduced the risk of SBIs (*p* = 0.005, *p* = 0.011, *p* = 0.014, *p* = 0.003, and *p* < 0.001, respectively). The LOS and rates of PICU admission were similar between the groups (Table 3).

In the fever-and-respiratory group, 241 infants were defined as having “clinical bronchiolitis” and 1553 were found to have “other respiratory symptoms”. In a sub-analysis of these groups, the risk of SBIs was lower in those with other respiratory symptoms compared to those with a fever only (5.5% vs. 7.5%, *p* = 0.029; OR (95% CI) = 0.72 (0.53–0.97)). The risk was further reduced in those with bronchiolitis (2.1%, *p* = 0.02; OR (95% CI) = 0.26 (0.11–0.64)). Five patients with bronchiolitis had SBIs—four cases of UTIs, out of which three were associated with the presence of RSV, and one case with *Enterobacter cloacae* bacteremia, despite negative viral test results and normal inflammatory markers. There were no cases of meningitis in the group of patients with clinical bronchiolitis. 

In the comparison between febrile infants screened for respiratory viruses with a positive and negative viral test result, the rate of SBIs was lower in infants with a confirmed viral infection (2.8% vs. 7% in the virus+ vs. virus− groups; *p* = 0.002, OR = 0.385, 95% CI = 0.203–0.728). In males, Jews, and infants with a CRP > 2 mg/dL, a positive virus test result was also associated with a lower risk of SBIs (*p* = 0.01, *p* = 0.028, and *p* = 0.001, respectively). As in patients with and without respiratory symptoms, the LOS and rates of PICU admission were similar among the groups (Table 4).

When examining the specific viruses, the most prevalent virus was RSV, for which 261 (21.2%) infants tested positive; of them, seven (2.7%) had SBIs. Influenza A was positive in 65 (6.1%) cases, and adenovirus was positive in 53 (4.6%) cases; of these infants, three (4.6%) and one (1.9%) had SBIs, respectively. Infants tested positive for parainfluenza, HMPV, and influenza B in thirty-four (3.1%), fifteen (1.4%), and six (0.6%) cases, respectively; none of them had SBIs. Out of 147 COVID tests, eleven were positive and one of them had SBIs. Rhinovirus, which was supplemented to the respiratory viral panel only during the final year of the study, caused a positive test in only 11 (40.7%) of the 27 cases, and none had SBIs.

In the first month of life, a higher prevalence of SBIs was observed in males compared to females (104/717 (14.5%) vs. 26/510 (5.1%), *p* < 0.001, odds ratio (OR) = 3.15, 95% confidence interval (CI) = 2.02–4.97). Similarly, higher rates of urinary tract infections (UTIs) were reported in males compared to females (92/670 (13.7%) vs. 16/469 (3.4%), *p* < 0.001, OR = 4.50, 95% CI = 2.61–7.75).

However, in the second month of life, a higher prevalence of SBIs was reported in females compared to males (35/823 (4.3%) vs. 25/1056 (2.4%), *p* = 0.021, OR = 1.83, 95% CI = 1.09–3.09). Similarly, higher rates of UTIs were observed in females compared to males (28/733 (3.8%) vs. 16/894 (1.8%), *p* = 0.012, OR = 2.18, 95% CI = 1.17–4.06).

**Figure 1:**
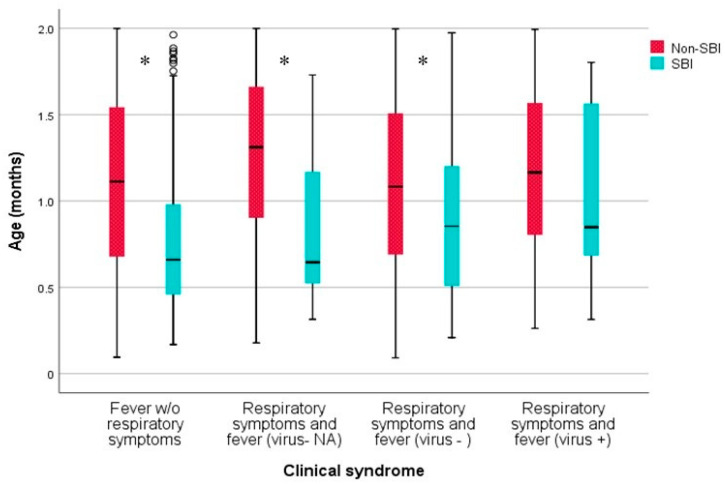
Median age of infants with and without SBIs in each group. SBI = serious bacterial infection; w/o = without. * statistically significant.

In the analysis of the age of infants in the different groups, infants with SBIs were younger than those without SBIs (*p* = 0.003), except in the virus+ group, for which the ages were similar (*p* = 0.278) (Figure 1).

## 4. Discussion

In this single-center retrospective study, we found that infants with a fever and respiratory symptoms had a lower risk of SBIs compared to infants with a fever only. Overall, the risk of SBIs was the lowest in infants with a positive viral test. We found several known risk factors for SBIs, such as the male gender, an age < 1 month, leukocytosis, and an elevated CRP; however, in conjunction with respiratory symptoms or a positive virus test, the risk of SBIs decreased.

In our study, the total rate of SBIs was 6.1% (7.5% in patients with a fever only and 5.1% in those with a fever and respiratory symptoms). The lowest rate (2.8%) was found in those with a positive respiratory virus test. Twelve infants with a positive virus test had SBIs, including ten with UTIs and two with IBIs—one case of bacteremia and one case of meningitis. The one patient that was defined as having meningitis based on a positive culture (*S. aureus*) had normal CSF indices, and was eventually discharged without antibiotics. In infants with bronchiolitis, the risk of SBIs was 75% lower than those with a fever only, with no cases of meningitis.

Similarly, several previous studies have found that the risk of SBIs is lower in infants with a positive virus test. In a retrospective cross-sectional study, the total rate of SBIs was 13.9%, and it was 77% lower in those with a documented viral infection. While the risk of bacteremia and UTIs was found to be lower, the number of cases of meningitis was too small for a difference to be detected [9]. In a multi-center prospective study, the rate of SBIs in influenza-positive patients was 2.5% and was significantly lower compared to influenza-negative patients. All SBI cases in influenza-positive patients were UTIs, with no cases of bacteremia or meningitis [10]. However, a prospective study showed an incidence of bacteremia of 1.1% and an incidence of meningitis of 0.8% in infants < 28 days old with positive viral infections, suggesting that the risk of IBIs in the younger age group is sufficiently high to warrant a full sepsis workup, regardless of the viral test result [11]. Another large prospective study showed that the risk of SBIs was 7% vs. 12.5% and the rate of UTIs was 5.4% vs. 10.1% in the RSV-positive vs. RSV-negative groups, respectively; however, in the youngest age group (<28 days), the risk of SBIs was substantial, and was not reduced by the presence of RSV [7]. Taken together, the risk of SBIs was found to be lower in the presence of a viral infection; however, the small percentage of patients with SBIs in this group is concerning. Some authors suggest that, in older infants (>28 days) with a confirmed viral infection, the necessity of a full sepsis workup (including a lumbar puncture (LP)) and hospitalization with empiric antibiotics should be re-considered. Due to the relatively high risk of UTIs, most authors recommend performing a urinalysis and urine culture, even in the presence of a viral infection [11].

In our study, the most common viruses were RSV (21.2%), influenza A (6.1%), and adenovirus (4.6%). In the study mentioned earlier, RSV was also the most common virus, detected in 55% of cases; parainfluenza resulted in a positive test in 17% of cases; and influenza A resulted in a positive test in 14% of cases [9]. An interesting theory presented by Greenfield et al. is that the frequency of SBIs may be higher in infants with systemic respiratory viruses (such as enterovirus or adenovirus, which may cause viremia) compared to those with viruses that remain in the respiratory mucosa (influenza, RSV, HMPV, and parainfluenza) [9]. We did not encounter cases of SBIs in patients with RSV, influenza A, or adenovirus, but the small numbers preclude any further conclusions.

The AAP committee supports the use of rapid influenza tests, which may allow for early detection and prompt antiviral treatment [12]. A potential reduction in the LOS was found in children hospitalized with respiratory symptoms when rapid on-site tests were adopted [13]. In a study conducted before multiplex viral testing became widely available, no difference in the LOS was found between febrile infants with and without a positive viral test [14]. A randomized controlled trial showed a trend towards a decreased LOS in pediatric inpatients when using viral testing, but the study was underpowered [15]. In our study, we did not have rapid viral testing. The LOS was not different between those with and without respiratory symptoms, or those with and without a confirmed viral infection.

In line with previous studies [16], we observed that male infants had a higher prevalence of SBIs compared to females within the first month of life. This can be attributed to a 4.5-fold greater risk of UTIs in males compared to females during this age. This may be attributed to factors such as the lack of circumcision or an elevated risk of UTIs following a recent circumcision [17]. However, in the second month of life, the risk is inverted, with females having a 2.2-fold greater risk of UTIs than males. Furthermore, the absence of an effect of respiratory symptoms on the risk of SBIs in females can be attributed to the generally lower rate of SBIs in females compared to males. As a result, the reduction rate due to respiratory symptoms was not statistically significant. It is possible that, with a larger cohort, this effect could be demonstrated.

We found that the male gender, an age < 1 month, leukocytosis, and an elevated CRP increased the risk of SBIs; however, in infants with these markers, the presence of respiratory symptoms decreased the risk of SBIs. In a retrospective study on infants with RSV, the presence of a fever, the absence of wheezing, and an age < 28 days increased the risk of a full sepsis workup, while the full workup increased the risk of hospitalization, antibiotic treatment, and a prolonged LOS [18].

The clinical appearance of infants is a critical initial step in identifying high-risk infants for SBIs, as evidenced by various scoring systems [19,20]. In the present study, the presence of respiratory symptoms did not impact the likelihood of SBIs in infants that appeared to be ill. However, it did influence the likelihood of SBIs in infants that appeared to be well. Nevertheless, relying solely on clinical appearance is inadequate for ruling out SBIs. Ultimately, the treatment of febrile infants aged >21 days is largely guided by the absence or presence of “positive inflammatory markers” [21]. The goal is to identify and promptly treat those with SBIs while avoiding invasive tests and treatments for those without SBIs [22]. Because of the unremarkable physical examination in most infants and the time spent pending culture results [2], inflammatory markers may aid in risk stratification and decision making [11]. Procalcitonin is considered more specific than other inflammatory markers, with a good discrimination for low-risk infants. However, this test is not universally available, and was also not available for our study [5,23]. The AAP guidelines recommend that, if procalcitonin is unavailable, the ANC, the CRP, and the peak of the temperature should be used [5,24]. In a recent study, using AAP’s low-risk criteria for 957 infants could reduce the LP and hospitalization with empiric antibiotics for nearly half of the cohort [23].

In recent years, new methods for rapid on-site viral testing have been developed for patients presenting to the ED with respiratory infections. These tests are highly sensitive and specific for diagnosing the presence of a viral infection within 20 min [25,26]. Additionally, to aid in clinical decision making, several diagnostic tests have been used in an attempt to distinguish between viral and bacterial infections. Srugo et al. examined a novel assay that integrates the CRP, tumor necrosis factor-related apoptosis-inducing ligand, and interferon γ induced protein-10. In a double-blind study, the assay showed a 93.8% sensitivity and an 89.8% specificity [27]. Despite the attractiveness of using these new methods, their high cost and limited availability preclude their widespread use, especially when resources are limited.

The major strength of our study is the amount of data acquisition for a large number of patients across over 20 years that was used to evaluate the effect of respiratory symptoms or viral testing on the risk of SBIs. Using a single tertiary center with similar attitudes towards admission and similar management policies eliminates site-to-site variability. The data acquisition was optimized using a computerized query tool, thus limiting acquisition error.

Our study had some limitations. Its retrospective nature limited us to data that were available in the medical records, and findings from a single tertiary hospital may not be generalizable to other institutions or the general population. A third of the infants with respiratory symptoms did not undergo viral testing. The database was lacking other data relevant to the study, including the status of RSV immunization and the overall antibiotic treatment during the hospital stay (as opposed to only the empiric treatment upon admission). Testing for procalcitonin is not routine in our center. We did not include an ANC analysis, as the study included infants who presented to the ED during night shifts when differential counts were not consistently available.

In conclusion, we found that the presence of respiratory symptoms, as well as a confirmed viral infection, decreased the risk of SBIs. The results are encouraging and give further reassurance to previous findings. However, given the small risk of SBIs even in patients with a positive viral infection, larger prospective studies are needed. The precise identification of low-risk infants is of utmost importance; a correct risk stratification will minimize the misclassification of patients with SBIs. Ultimately, the correct identification of low-risk infants in whom a fever is attributable to a viral infection may change guidelines and bring a new phase in the management of neonatal fevers.

## Figures and Tables

**Table 1:** Clinical outcomes of the different groups of febrile infants.

	Fever Only (n = 1312)	Fever and Resp, Virus (−) (n = 759)	Fever and Resp, Virus (+) (n = 427)	Fever and Resp, Virus NA (n = 608)	*p*-Value
SBI	99 (7.5) Reference	53 (7.0) *p*-value * = 0.516	12 (2.8) *p*-value * = 0.001 OR = 0.35 95% CI = 0.19–0.65	26 (4.3) *p*-value * = 0.005 OR = 0.49 95% CI = 0.29–0.81	0.001
IBI Bacteremia Meningitis	21 (1.6) 20 (1.6) 6 (1.6)	14 (1.8) 12 (1.6) 3 (1.0)	2 (0.5) 1 (0.3) 1 (0.9)	9 (1.5) 9 (1.7) 1 (0.9)	0.282 0.247 NA
UTI	83 (6.8) Reference	40 (5.5) *p*-value * = 0.203	10 (3.0) *p*-value * = 0.010 OR = 0.43 95% CI = 0.22–0.83	19 (3.8) *p*-value * = 0.017 OR = 0.49 95% CI = 0.27–0.89	0.014

Numbers are presented as absolute numbers of cases and percentages. SBI = serious bacterial infection; IBI = invasive bacterial infection; UTI = urinary tract infection; resp = respiratory; virus (−) = virus-negative; virus (+) = virus-positive; NA = not available. * Compared to the reference (fever only).

**Table 2:** Univariate and multivariate analyses of risk factors for SBIs.

	P Uni (χ^2^ Test)	P Multi (Binary Regression)	Adjusted OR (95% CI)
Gender—male	0.002	0.056	1.49 (0.99–2.25)
Age < 1 month	0.000	0.000	2.74 (1.83–4.10)
WBC > 15,000	0.000	0.000	2.64 (1.74–3.99)
CRP > 2 mg/dL	0.000	0.000	4.78 (3.19–7.15)
Fever w/o respiratory	0.004	0.001	1.92 (1.29–2.85)

Uni = univariate analysis; multi = multivariate analysis; WBC = white blood cell count; CRP = C-reactive protein; w/o = without.

**Table 3:** Comparison of febrile infants with and without respiratory symptoms.

	Fever Only Total = 1312 N (%)		Fever and Respiratory Total = 1794 N (%)		*p*-Value *	OR (95% CI)
	No SBI N (%)	SBI N (%)	No SBI N (%)	SBI N (%)		
Whole cohort	1213 (92.5)	99 (7.5)	1703 (94.9)	91 (5.1)	0.004	0.65 (0.49–0.88)
Gender, male	673 (90.7)	69 (9.3)	971 (94.2)	60 (5.8)	0.005	0.60 (0.42–0.86)
Gender, female	540 (94.7)	30 (5.3%)	732 (95.9)	31 (4.1)	0.300	
Age < 1 m	487 (87)	73 (13)	610 (91.5)	57 (8.5)	0.011	0.62 (0.43–0.9)
Age 1–2 m	726 (96.5)	26 (3.5)	1093 (97)	34 (3)	0.595	
Ethnicity, Jew	717 (92.2)	61 (7.8)	1085 (94.9)	58 (5.1)	0.014	0.63 (0.43–0.91)
Ethnicity, Arab	482 (94.9)	26 (5.1)	399 (93)	30 (7)	0.228	
WBC < 15,000/μL ^a^	877 (95)	46 (5)	1232 (96)	51 (4)	0.254	
WBC > 15,000/μL	185 (78.7)	50 (21.3)	236 (88.4)	31 (11.6)	0.003	0.49 (0.30–0.74)
CRP < 2 mg/dL ^b^	366 (95.8)	16 (4.2)	872 (96)	36 (4)	0.852	
CRP > 2 mg/dL	87 (65.4)	46 (34.6)	253 (88.5)	33 (11.5)	<0.001	0.25 (0.15–0.41)
Appears ill	63 (91.3)	6 (8.7)	99 (92.5)	8 (7.5)	0.770	
Appears well	981 (92.1)	84 (7.9)	1571 (95)	83 (5)	0.002	0.62 (0.45–0.84)
Discharge	218 (93.2)	16 (6.8)	330 (97.1)	10 (2.9)	0.027	0.41 (0.13–0.93)
Hospitalization	995 (92.3)	83 (7.7)	1373 (94.4)	81 (5.6)	0.031	0.70 (0.51–0.97)
LOS (days)	3.6 ± 4.9	3.1 ± 2.5	3.4 ± 4.4	3.3 ± 3.2	0.540	
PICU admission	68 (5.6)	4 (4)	80 (4.7)	3 (3.3)	0.393	

SBI = serious bacterial infection; m = months; WBC = white blood cell count; CRP = C-reactive protein; LOS = length of stay; PICU = pediatric intensive care unit. * Risk of SBIs by respiratory symptoms (yes/no) for each variable. ^a^ For CBC, n = 2708. ^b^ For CRP, n = 1709.

**Table 4:** Comparison of febrile infants with respiratory symptoms with and without a documented viral infection.

	Fever and Resp Virus (−) Total = 759 N (%)		Fever and Resp Virus (+) Total = 427 N (%)			
	No SBI N (%)	SBI N (%)	No SBI N (%)	SBI N (%)	*p*-Value *	OR (95% CI)
Whole cohort	706 (93)	51 (7)	415 (97.2)	12 (2.8)	0.002	0.39 (0.2–0.73)
Gender, male	408 (92.1)	35 (7.9)	232 (97.1)	7 (2.9)	0.01	0.35 (0.15–0.80)
Gender, female	298 (94.3)	18 (5.7)	183 (97.3)	5 (2.7)	0.14	
Age < 1 m	291 (90.4)	31 (9.6)	155 (95.7)	7 (4.3)	0.041	0.42 (0.18–0.99)
Age 1–2 m	415 (95)	22 (5)	260 (98.1)	5 (1.9)	0.036	0.36 (0.14–0.97)
Ethnicity, Jews	491 (93.5)	34 (6.5)	249 (97.3)	7 (2.7)	0.028	0.40 (0.18–0.93)
Ethnicity, Arabs	158 (92.4)	13 (7.6)	136 (96.5)	5 (3.5)	0.126	
WBC < 15,000/μL ^a^	542 (94.4)	32 (5.6)	293 (97.7)	7 (2.3)	0.028	0.40 (0.18–0.93)
WBC > 15,000/μL	124 (87.3)	18 (12.7)	42 (93.3)	3 (6.7)	0.266	
CRP > 2 mg/dL ^b^	504 (95.5)	24 (4.5)	206 (96.7)	7 (3.3)	0.438	
CRP > 2 mg/dL	116 (82.9)	24 (17.1)	84 (97.7)	2 (2.3)	0.001	0.12 (0.03–0.50)
Appears ill	24 (85.7)	4 (14.3)	44 (93.6)	3 (6.4)	0.413	
Appears well	678 (93.3)	49 (6.7)	366 (97.6)	9 (2.4)	0.002	0.34 (0.17-0.70)
Discharge	132 (96.4)	5 (3.6)	71 (98.6)	1 (1.4)	NA	
Hospitalization	574 (92.3)	48 (7.7)	344 (96.9)	11 (3.1)	0.004	0.38 (0.2-0.75)
LOS (days)	3.3 ± 4.4	3.5 ± 3.5	3.6 ± 4.4	2.8 ± 1.4	0.541	
PICU admission	31 (4.4)	1 (1.9)	23 (5.5)	1 (8.3)	0.764 **	

Virus (−) = virus-negative; virus (+) = virus-positive; SBI = serious bacterial infection; WBC = white blood cell count; CRP = C-reactive protein; LOS = length of stay; PICU = pediatric intensive care unit. * Risk of SBI by virus result (positive/negative) for each variable. ** Fisher exact test. ^a^ For CBC, n = 1061. ^b^ For CRP, n = 967.

## Data Availability

Data is available by request from the corresponding author.

## Supplementary Materials

The following supporting information can be downloaded at: https://www.mdpi.com/article/10.3390/jcm12144636/s1, Table S1: Comparison of febrile infants younger than 28 days of age.
