# Supplementary material for: The Impact of Respiratory Symptoms on the Risk of Serious Bacterial Infection in Febrile Infants < 60 Days Old
Source: J Clin Med. 2023 Jul 12;12(14):4636. doi: 10.3390/jcm12144636 (PMC10380775; doi:10.3390/jcm12144636)
Supplement: Supplementary file 1 [file jcm-12-04636-s001.zip › jcm-2445871-supplementary.pdf]

Table S1. Comparison of febrile infants younger than 28 days of age

	**Fever only**	**Fever & Resp, virus (NA)**	**Fever & Resp, virus (-)**	**Fever & Resp, virus (+)**
week 1	5/45 (11.1%)	NA	1/23 (4.3%)	NA
week 2	18/133 (13.5%)	2/17 (11.8%)	12/81 (14.8%)	2/31 (6.5%)
week 3	36/242 (14.9%)	9/44 (20.5%)	14/153 (9.2%)	3/65 (4.6%)
week 4	14/140 (10%)	4/64 (6.3%)	8/123 (6.5%)	2/66 (3%)
Total	73/560 (13%)	15/125 (12%)	35/380 (9.2%)	7/162 (4.3%)
p-value	0.565	0.083	0.189	0.733

virus (-) =virus negative; virus (+) =virus positive
